# Sigmoidal kinetics define porcine intestinal segregation of electrogenic monosaccharide transport systems as having multiple transporter population involvement

**DOI:** 10.14814/phy2.14090

**Published:** 2019-05-06

**Authors:** Marina Subramaniam, Cole B. Enns, Matthew E. Loewen

**Affiliations:** ^1^ Department of Veterinary Biomedical Sciences Western College of Veterinary Medicine University of Saskatchewan Saskatoon Saskatchewan Canada

**Keywords:** Gastrointestinal tract, glucose absorption, porcine, sigmoidal, sodium‐dependent glucose transporter (SGLT)

## Abstract

Kinetic characterization of electrogenic sodium‐dependent transport in Ussing chambers of d‐glucose and d‐galactose demonstrated sigmoidal/Hill kinetics in the porcine jejunum and ileum, with the absence of transport in the distal colon. In the jejunum, a high‐affinity, super‐low‐capacity (Ha/sLc) kinetic system accounted for glucose transport, and a low‐affinity, low‐capacity (La/Lc) kinetic system accounted for galactose transport. In contrast, the ileum demonstrated a high‐affinity, super‐high‐capacity (Ha/sHc) glucose transport and a low‐affinity, high‐capacity (La/Hc) galactose transport systems. Jejunal glucose transport was not inhibited by dapagliflozin, but galactose transport was inhibited. Comparatively, ileal glucose and galactose transport were both sensitive to dapagliflozin. Genomic and gene expression analyses identified 10 of the 12 known SLC5A family members in the porcine jejunum, ileum, and distal colon. Dominant SGLT1 (SLC5A1) and SGLT3 (SLC5A4) expression was associated with the sigmoidal Ha/sLc glucose and La/Lc galactose transport systems in the jejunum. Comparatively, the dominant expression of SGLT1 (SLC5A1) in the ileum was only associated with Ha glucose and La galactose kinetic systems. However, the sigmoidal kinetics and overall high capacity (Hc) of transport is unlikely accounted for by SGLT1 (SLC5A1) alone. Finally, the absence of transport and lack of pharmacological inhibition in the colon was associated with the poor expression of SLC5A genes. Altogether, the results demonstrated intestinal segregation of monosaccharide transport fit different sigmoidal kinetic systems. This reveals multiple transporter populations in each system, supported by gene expression profiles and pharmacological inhibition. Overall, this work demonstrates a complexity to transporter involvement in intestinal electrogenic monosaccharide absorption systems not previously defined.

## Introduction

Sodium‐dependent glucose absorption along the small intestine (jejunum and ileum) of mammals (rats, pigs, rabbits, and humans) represent a heterogeneous transport system (Brot‐Laroche et al. 1988; Malo 1990; Koepsell and Spangenberg 1994). In particular, this has been studied extensively in porcine models that have diets similar to humans and a comparable gastrointestinal tract anatomy and physiology (Halaihel et al. 1999; Aschenbach et al. 2009). This heterogeneous system is defined by the presence of two types of sodium‐dependent glucose transport kinetics in the jejunum and ileum (Stumpel et al. 2001; Wright 2001; Wright et al. 2011). Specifically, the transport kinetics of glucose in the jejunum have been identified as a high‐affinity, low‐capacity (Ha/Lc) transport system, while the ileum demonstrates a low‐affinity, high‐capacity (La/Hc) glucose transport system (Brot‐Laroche et al. 1986; Ferraris and Diamond 1986; Wolffram et al. 1989; Arai et al. 1995; Halaihel et al. 1999; Diez‐Sampedro et al. 2001; Wood and Trayhurn 2003; Aschenbach et al. 2009; Herrmann et al. 2012; Wright 2013). These systems were characterized with Michaelis–Menten kinetics (hyperbolic saturation), suggesting only one transporter involvement in each segment. (Kaunitz and Wright 1984; Brot‐Laroche et al. 1988; Wolffram et al. 1989; Halaihel et al. 1999). However, the small number of incremental increases in the glucose gradient used may have limited generating precise kinetic fits (Herrmann et al. 2012).

Thus far, these heterogeneous Michaelis–Menten systems were thought to be the product of the Ha/Lc sodium‐dependent glucose transporter 1 (SGLT1), a member of the SLC5A (solute carrier family member 5A) transporter family (Wood and Trayhurn 2003; Wright et al. 2011; Harada and Inagaki 2012; Wright 2013; Poulsen et al. 2015). The SLC5A family is responsible for the cotransport of glucose and sodium, resulting in a measurable current (Wood and Trayhurn 2003; Wright et al. 2011; Harada and Inagaki 2012; Wright 2013; Poulsen et al. 2015). Recently, porcine jejunal and ileal sodium‐dependent glucose transport kinetics revealed Ha kinetics in both segments, suggesting SGLT1 (SLC5A1) may be responsible in both segments (Herrmann et al. 2012). However, it was concluded that the Hc kinetics observed in the ileum cannot be explained by SGLT1 (SLC5A1) (Herrmann et al. 2012).

The possibility of a modified SGLT1 (SLC5A1) transporter responsible for the segregation of sodium‐dependent glucose transport in the jejunum and ileum has been studied by Herrmann et al. (2012), as well as Klinger et al. (2018). It was concluded that the possibility of glycosylation‐ or phosphorylation‐mediated changes to the SGLT1 (SLC5A1) transporter may compensate in situations where glucose transport is decreased, but it was not the reason for the heterogeneity of transport between the jejunum and ileum (Klinger et al. 2018). Additionally, kinetic analysis only demonstrated Michaelis–Menten kinetics, suggesting only one transporter involvement (Herrmann et al. 2012). However, the presence of a modified SGLT1 should have likely created sigmoidal kinetics.

Here, we report the first detailed kinetic description of both sodium‐dependent glucose and galactose transport systems along the porcine gastrointestinal tract, with use of the dapagliflozin inhibitor and an extensive gene expression analysis of all known pig SLC5A orthologs. Overall, this description best fit sigmoidal/Hill kinetics, indicating the involvement of multiple populations of transporters in each segment. Additionally, different functional populations of transporters are present between the jejunum and ileum.

## Materials and Methods

### Animals

Thirteen 7‐ to 9‐week‐old purebred Yorkshire barrows were housed in pairs and provided with a commercial, non‐medicated diet and water ad libitum. Pigs were housed in the Animal Care Unit at the University of Saskatchewan, and maintained in accordance with the guidelines of the Canadian Council on Animal Care (Care CCoA, 2005). All animal protocols were approved by the Animal Care Committee at the University of Saskatchewan (AUP#: 20130034).

### Ex vivo tissue collection

Pigs were euthanized and the gastrointestinal tract was removed. The gastrointestinal tract was separated to obtain sections of the jejunum, ileum, and distal colon. The jejunum was obtained 18 inches distal from the stomach, the ileum was sampled 2 inches proximal of the ileal–caecal junction, and the distal colon was sampled 4 inches proximal of the rectum.

### Electrophysiology

#### Ussing chamber technique

Porcine intestinal samples were examined in Ussing chambers using techniques adapted from our group (Tarran et al. 2002; Loewen et al. 2003, 2004). The Ussing chamber system used in this study was an EasyMount Ussing Chamber System (Physiologic Instruments Inc., San Diego, CA). Each segment was placed in a modified Krebs buffer containing: 114 mmol/L NaCl, 5 mmol/L KCl, 2.15 mmol/L CaCl_2_·2H_2_O, 1.1 mmol/L MgCl_2_·6H_2_O, 0.3 mmol/L NaH_2._PO_4·_H_2_O, 1.65 mmol/L Na_2_HPO_4_, and 25 mmol/L NaHCO_3_ at pH 7.4, and devoid of glucose. The lumen of the intestine was washed with buffer using a syringe and 18‐gauge needle to clean the luminal membrane of any residual chyme. The serosa and longitudinal muscle layers were removed from the intestinal samples with forceps prior to mounting on 1‐cm^2^ Ussing chamber inserts (Slider Number: P2314, Physiologic Instruments Inc., San Diego, CA). Overall, the intestinal segments were stripped and mounted within 20 min from time of euthanasia. Once mounted, tissues were inserted into the Ussing chamber, and both apical and basolateral surfaces were bathed with 5 mL of Krebs buffer solution. The chambers were continuously gassed for the duration of the experiment with 5% CO_2_ and 95% O_2_ (Clarke 2009). Needle valves were also present for the adjustment of gas flow into the chambers.

The transepithelial voltage and passing current set across the tissue was measured via 3 mol/L KCl agar bridges and Ag/AgCl reference electrodes. These electrodes were connected to leads that lead to the voltage/current clamp. The short‐circuit current (Isc) was measured by the software program LabChart (ADInstruments Pty Ltd.) in microamperes (*μ*A), and this was a result of the tissue current opposing the current induced by the electrodes. The tissues were pulsed every 30 sec at 0.001 V to determine tissue resistances from the resulting current. A recirculating water bath capable of chilling and heating was responsible for maintaining the buffer in the Ussing chambers at 36–37°C.

The glucose gradients used were: 1–25, 30, 40, and 50 mmol/L in sequential order. The gradient from 1–25 mmol/L was added to the chamber in increasing 1 mmol/L increments, followed by increments of 30, 40, and 50 mmol/L. The tissues were allowed to reach a steady baseline current for 20 min prior to the addition of d‐glucose to the apical side of the chamber. An equivalent amount of d‐mannitol was added to the basal side to prevent the development of an osmotic gradient from the addition of glucose. Furthermore, mannitol would not interfere with glucose transport as mannitol is unable to transport across the intestinal epithelium. Prior to the addition of each increment of glucose and mannitol, a wait period of 3–4 min was allowed between each increment for the current to reach a steady state. The experiment with d‐galactose was conducted in a similar manner.

#### Chemicals

For pharmacological characterization, 0.001, 0.01, 0.1, 1, 10, 100, 200, and 300 *μ*mol/L of dapagliflozin (AdooQ^®^ Bioscience, Irvine, CA) was used in sequential order in the Ussing Chamber as final concentrations. The drug was added to the apical side, after the jejunum, ileum, and distal colon were at final glucose/galactose and mannitol concentrations of 50 mmol/L. A wait period of 5–7 min was allowed between each increment for the current to reach steady state.

#### RNA extraction and cDNA synthesis using RT‐qPCR

Approximately 1 mg samples of tissue were obtained from the dissection of the intestinal tract and stored in RNA*later* ^®^ RNA Stabilization Solution (Thermo Fisher Scientific, Baltics) at −80°C. RNA was isolated using the TRIzol reagent (Ambion *Life* Technologies, Van Allen Way, CA), and cDNA was synthesized through reverse transcription PCR using the qScript™ cDNA SuperMix (Quanta Biosciences, Maryland, USA) according to the manufacturer's protocol. The cDNA samples were stored at −80°C for subsequent use in quantitative PCR.

#### Genomic identification of SLC5A genes

A detailed BLAST+ (Basic Local Alignment Search Tool) application was used to identify all of the annotated porcine SLC5A transporters. The 12 human SLC5A transporter sequences retrieved from the National Center for Biotechnology Information (NCBI) website (http://www.ncbi.nlm.nih.gov/) were used in a “blastn” command to search for similar mRNA sequences in the porcine genome (Wright 2013). The expect value (*e*‐value) was used to assess the significance of the match, where an e‐value close to zero was considered significant and an *e*‐value higher than 10^−15^ was considered as a nonsignificant match. Once SLC5A transporters were identified, phylogenic trees were generated using the alignment program CLUSTAL W and MEGA7 (www.megasoftware.net) software.

#### Gene transcript expression levels by quantitative polymerase chain reaction

Quantitative PCR was performed by RT‐qPCR reaction using the GoTaq^®^qPCR Master Mix containing SYBR^®^Green1 (Promega, Madison, USA). The Bio‐Rad QPCR system (CFX96™ Bio‐Rad Laboratories, Inc) was used to perform these reactions. The total volume of the PCR reaction was 12.5 *μ*L. A total of 40 cycles of qPCR was performed, with each cycle consisting of GoTaq^®^ Hot Start Polymerase activation at 95°C for 2 min, and then denaturation at 95°C for 15 sec, followed by annealing/extension at the primer's hybridization temperature for 1 min. The dissociation step was at 60–95°C. Serial dilutions of cDNA were also used to generate a standard curve for each target gene, where the efficiency of each primer was calculated. The efficiencies for the primers ranged from 1.8 to 2.0. Porcine *β*‐actin was used as the housekeeping gene and was used to normalize the relative mRNA expression levels of SGLTs in the pig. The method of analysis used to represent the relative expression levels was calculated using delta CT, where the amount of gene of interest was normalized to the amount of *β*‐actin in that sample (Sasaki et al. 2010).

The primers for the porcine SLC5A genes and housekeeping gene were designed through the Integrated DNA Technologies website (https://www.idtdna.com/site), where the sequences and their accession numbers are presented in Table 1.

**Table 1:** Pig primer sequences used for quantitative PCR

Gene	Forward primer (5′‐3′)	Reverse primer (5′‐3′)	Accession number
SGLT1	CTGTGGTGTGCACTACTT	GGTCAATACGCTCCTCTTT	NM_001164021.1
SGLT2	CTGGGCTGGAACATCTAC	CGTAACCCATGAGGATGAA	XM_021086375.1
SMIT1	TCTGAGATGCAGTGAGAATAG	CTGAATGACCCAAGGAAGA	XM_005657149.3
SGLT3	ATTGTGTGGGTCCCATTAG	GAAGGCTCCCTGTTCATT	NM_214182.1
NIS	GTTAGATCCTCTCTGGACAAC	CCACCTGTAAAGTGGGTATAG	NM_214410.1
SMVT	CAGCAATCAGCATCTTTGG	GGCCATGTTGGTCACTAT	XM_013996254.2
CHT	AGCAGTTGCAGGTGTAATA	GTAGACACACTCAGAGGAAC	XM_021087111.1
SMCT1	CCTCACTGCCAGCTTTAT	TCGTAGGTGCTGGTAAATC	NM_001291414.1
SGLT4	GGAACCTTTACCTCTCCAC	CTCCTGAAAGCCCAGAAA	XM_021096677.1
SGLT5	TCCAGATCGAGAACCTCA	AGAAGAGGTTGACACACAC	NM_001012297.1
SMIT2	CCCTCACCTCCATCTTTAAC	TGGAGACCAGCACTAGAA	NM_001110422.1
SMCT2	CACTGGGTAAGGAGGAAAG	CAGGTGTGAGCTGAATGA	XM_003122908.4
*β*‐ACTIN	CACCACTGGCATTGTCAT	GTGGTGGTGAAGCTGTAG	XM_021086047

#### Designating Affinities (Ha or La) and Capacities (sHc, Hc, sLc, or Lc)

After the *K*_m_ and *V*_max_ values were calculated from Hill/sigmoidal fits (preferred model) using GraphPad Prism 8 (GraphPad Software, Inc.), the affinities, Ha or La, and the capacities, super‐high‐capacity (sHc), Hc, super‐low‐capacity (sLc), and Lc were designated to the transport systems. To denote whether a system was Ha or La, the *K*_m_ values were statistically compared between the jejunum and ileal glucose and galactose transport. If *K*_m_ values were low and nonsignificant from each other, they were characterized as Ha. In Tables 2 and 3, the *K*_m_ values for the jejunum and ileum glucose transport were low and nonsignificant from each other, which designated as Ha. If *K*_m_ values were higher and significantly different from the low *K*_m_ values, then those were designated as La. In Tables 2 and 3, the *K*_m_ values for the jejunum and ileum galactose transport were nonsignificant from each other and higher than the corresponding glucose transport, thus they were designated as La. Similarly, if the *V*_max_ values were low and significantly different from the high *V*_max_ values, then those were denoted as low‐capacity, Lc (Tables 2 and 3). Additionally, if the low *V*_max_ values were significantly different from each other, then it was further designated as Lc and sLc (Tables 2 and 3). If the *V*_max_ values were high, and significantly different from the low *V*_max_ values, then those would be designated as Hc (Table 2). Additionally, if the high *V*_max_ values were significantly different from each other, then they were further designated as Hc and sHc (Tables 2 and 3). Altogether in this study, the characterization of Ha/sLc, La/Lc, Ha/sHc, and La/Hc were determined by statistically comparing *K*_m_ and *V*_max_ values between monosaccharide transport and segments.

**Table 2:** *V*_max_ and *K*_m_ values for d‐glucose and d‐galactose electrogenic absorption

d‐glucose				d‐galactose				Glucose versus galactose	
Tissue	*K*_m_ ± SEM (mmol/L)	*V*_max_ ± SEM (*μ*A/cm^2^)	Hill slope	Tissue	*K*_m_ ± SEM (mmol/L)	*V*_max_ ± SEM (*μ*A/cm^2^)	Hill slope	*K*_m_	*V*_max_
Jejunum	4.6 ± 0.4^**A**^	11.1 ± 2.3^**A**^	4.3 ± 0.5	Jejunum	19.1 ± 1.2^**A**^	30.0 ± 5.0^**A**^	11.5 ± 1.8	*	*
Ileum	6.3 ± 0.9^**A**^	100.7 ± 9.3^**B**^	3.1 ± 0.6	Ileum	17.4 ± 2.1^**A**^	60.3 ± 7.1^**B**^	2.7 ± 0.9	*	*
Distal Colon	nd	nd	nd	Distal colon	nd	nd	nd	nd	nd

*V*_max_ (*μ*A) and *K*_m_ (mmol/L) represent sodium‐dependent electrogenic d‐glucose and d‐galactose absorption for each tissue type in pig. ND represents nondetectable transport of those substrates. The area of intestinal segment was 1 cm^2^. Data are presented as means ± SEM. The same letter superscripts for *K*_m_ values between the jejunum and ileum in response to d‐glucose and d‐galactose transport indicates no significant differences. Different letter superscripts for the *V*_max_ values between the jejunum and ileum in response to d‐glucose and d‐galactose transport represents significance (Student's *t*‐test, *P* < 0.05). Single asterisk represents significant difference between jejunal *K*_m_ and *V*_max_ values for d‐glucose and d‐galactose transport (Student's t‐test, *P* <  0.05). Single asterisk represents significant difference between ileal *K*_m_ and *V*_max_ values for d‐glucose and d‐galactose transport (Student's t‐test, *P* <  0.05).

**Table 3:** Final designations of transport systems for each segment

Tissue	d‐glucose	d‐galactose
Jejunum	High‐affinity, super‐low‐capacity (Ha/sLc)	Low‐affinity, low‐capacity (La/Lc)
Ileum	High‐affinity, super‐high‐capacity (Ha/sHc)	Low‐affinity, high‐capacity (La/Hc)
Distal colon	ND	ND

Kinetic transport systems defined for each porcine intestinal segment for d‐glucose and d‐galactose sodium‐dependent transport. These designations are further defined in the Material and Methods Section. ND represents nondetectable kinetic characterization of sodium‐dependent transport.

#### Statistical analyses

d‐glucose and d‐galactose gradients in the jejunum and ileum demonstrated sigmoidal saturation kinetics, with Hill coefficients >1 (Table 2). This was determined from conducting an *F*‐test between the traditionally used Michaelis–Menten fit and a sigmoidal/Hill fit to confirm the preferred model using GraphPad Prism 8 (GraphPad Software, Inc.) (Kuehl et al. 2005; Obi et al. 2011). The sigmoidal/Hill fit was determined as the preferred model, with a *P* < 0.001. Generally, sigmoidal/Hill fits indicate the presence of more than one binding site/transporter, with Hill coefficients usually >1 (Kekuda et al. 1999; Miyauchi et al. 2010). The *V*_max_ (represented in *μ*A/cm^2^) and *K*_m_ (represented in mmol/L) values for glucose and galactose transport were calculated using the equation for “One Site‐Specific Binding with Hill slope” for the jejunum and ileum using GraphPad Prism 8. The equation used for the sigmoidal Hill kinetics was:

$$\text{Sigmoidal Hill fit}:Y=V_{\max}X^{h}/\left({\text{Kd}}^{h}+X^{h}\right)$$

All data met parametric assumptions, being normally distributed and exhibited homogeneity of variance. A Student's *t*‐test was performed between jejunum and ileum *V*_max_ and *K*_m_ values for glucose and galactose to determine significance (Zar 2010). Similarly, a Student's *t*‐test was performed between jejunal glucose and galactose transport for *V*_max_ and *K*_m_, as well as ileal glucose and galactose transport for *V*_max_ and *K*_m_ to determine significance. A *P* < 0.05 was considered to be significantly different.

For the inhibitor dapagliflozin, the percent activity remaining was calculated from the division of the 50 mmol/L increment Isc (the concentration where the transporter is 100% saturated and it is 100% uninhibited) and the resulting drop in current from the addition of each drug concentration. These calculations were performed for both glucose and galactose inhibitor data. The *K*_*i*_ values for the dapagliflozin response were calculated for the jejunum and ileum using the “One‐Site Fit logIC50” from GraphPad Prism 8 (GraphPad Software, Inc.). This fit follows the equation: *Y* = Bottom + (Top‐Bottom)/(1 + 10^(*X* − LogIC50)^). Parametric one‐way ANOVA's were performed on the three intestinal sections to determine significance for inhibition. After ANOVA, pairwise comparisons were made using Tukey's posteriori tests, as appropriate. A Student's *t*‐test was performed between *K*_*i*_ values to determine significance. For the qPCR analyses, relative expressions were determined by the Bio‐Rad system, where the threshold cycle was established using the housekeeping gene, *β*‐actin. A two‐way ANOVA (two factors: intestinal segment and gene) was conducted on the gene expressions. After ANOVA, pairwise comparisons were made using Tukey's posteriori tests, as appropriate. All statistical analyses were performed on SigmaPlot (Systat Software Inc., San Jose, CA).

## Results

### Characterization of kinetic transport systems

#### Jejunum – Ha/sLc glucose and La/Lc galactose kinetic transport systems

**Figure 1:**
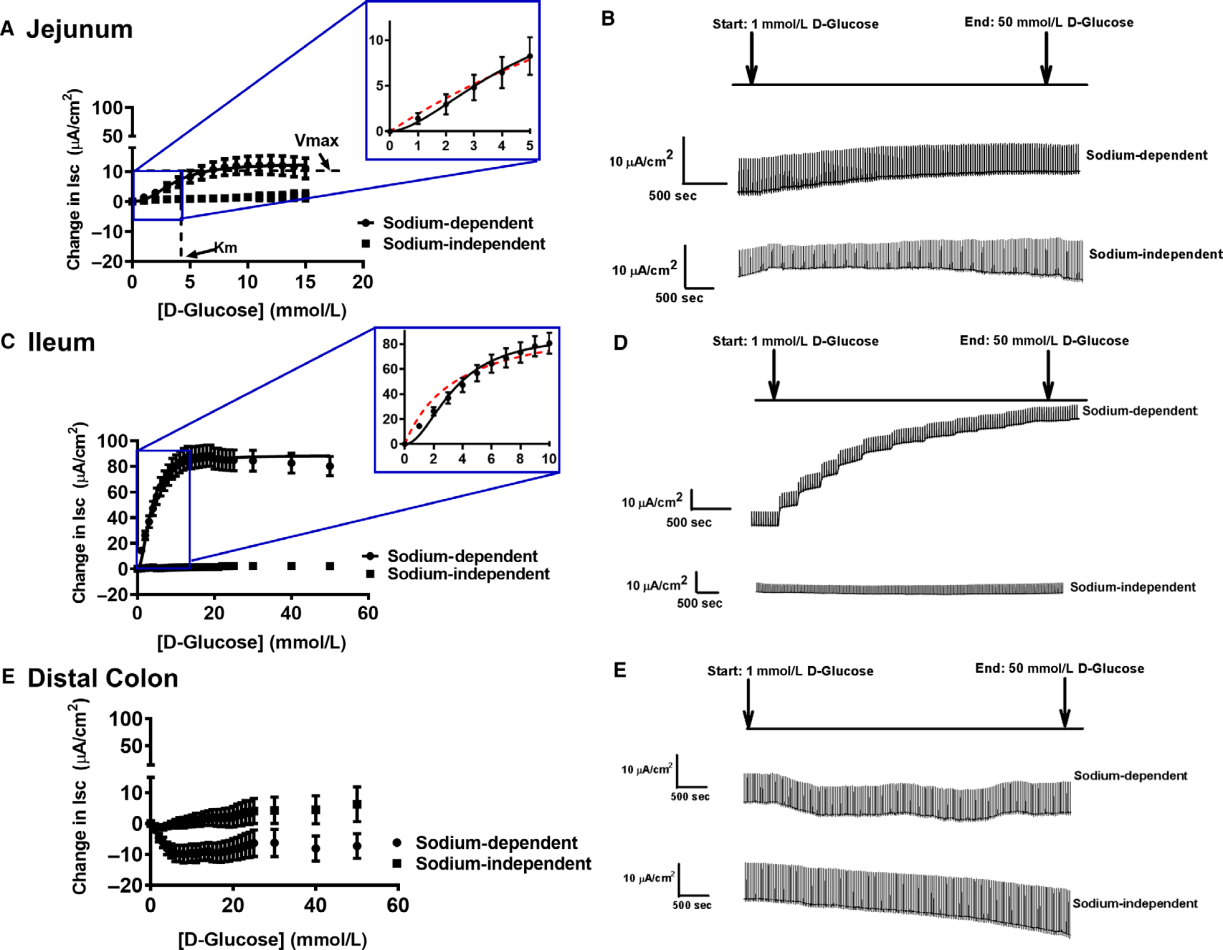
Sigmoidal kinetics for sodium‐dependent electrogenic glucose transport in the (A) jejunum, with the inset representing exemplary fits for Michaelis–Menten (red dotted line) and sigmoidal/Hill kinetics (black solid line). The representative traces are illustrated on the right side (B) (sodium‐dependent: Pig *N* = 11; sodium‐independent: pig *N* = 3). (C) Sigmoidal kinetics for the ileum, with the inset representing exemplary fits for Michaelis–Menten (red dotted line) and sigmoidal/Hill kinetics (black solid line). The representative traces are illustrated on the right side (D) (sodium‐dependent: pig *N* = 13; sodium‐independent: pig *N* = 3). (E) No kinetics observed in the distal colon, illustrated with representative traces on the right side (F) (sodium‐dependent: Pig *N* = 13; sodium‐independent: pig *N* = 3). The *V*_max_ and *K*_m_ is represented in the (A) jejunum for illustration purposes. Data are represented as means ± SEM.

**Figure 2:**
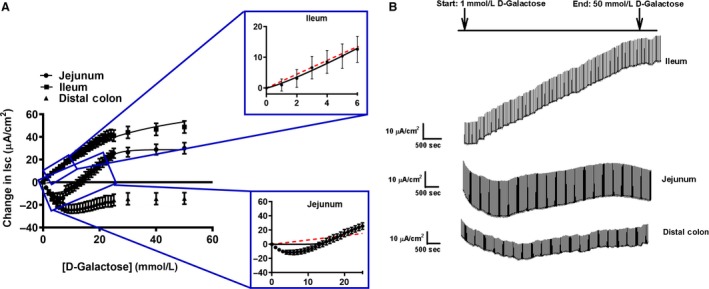
Sigmoidal kinetics for sodium‐dependent electrogenic galactose transport in the (A) jejunum, ileum, and colon. The inset for the jejunum (top) and ileum (bottom) represents exemplary fits for Michaelis–Menten (red dotted lines) and sigmoidal/Hill kinetics (black solid line). The representative traces are illustrated on the right side (B) (pig: jejunum: *N* = 5, ileum: *N* = 6, distal colon: *N* = 6). Data are represented as means ± SEM.

The electrogenic sodium‐dependent glucose and galactose transport followed sigmoidal/Hill kinetics in the porcine jejunum, with Hill coefficients >1 (Table 2, Figs. 1A and 2A). An *F*‐test was conducted to confirm that the sigmoidal/Hill fit was preferred over a Michaelis–Menten, hyperbolic fit (*P* < 0.001). Additionally, exemplary sigmoidal/Hill and Michaelis–Menten fits for the smaller substrate increments are presented in the insets for Figures 1A and 2A. These insets represent the difference between both models, demonstrating the sigmoidal/Hill model is a better fit. Jejunal glucose transport became saturated around 14–15 mmol/L glucose and resulted in very low *V*_max_ and *K*_m_ values (Table 2 and Fig. 1A). In contrast, jejunal glucose transport revealed a significantly lower *V*_max_ compared to the galactose transport system (Table 2, *P* < 0.001). The *K*_m_ values between jejunal glucose and galactose transport revealed significantly higher *K*_m_ in jejunal galactose transport (Table 2, *P* < 0.001). Thus, these label a Ha/sLc (low *K*_m_, very low *V*_max_) glucose transport system and a La/Lc (high *K*_m_, low *V*_max_) galactose transport system in the porcine jejunum (Tables 2 and 3). Sodium dependency of glucose transport in the jejunum was demonstrated by replacement of sodium with choline creating a sodium‐free Krebs buffer, which eliminated the glucose‐mediated current (Fig. 1A and B) (Delezay et al. 1995).

#### Ileum – Ha/sHc glucose and La/Hc galactose kinetic transport systems

In the porcine ileum, glucose and galactose gradients followed sigmoidal/Hill kinetics for both substrates, with Hill coefficients >1 (Table 2, Figs. 1C and 2A). An *F*‐test was conducted to confirm that the sigmoidal/Hill fit was preferred over a Michaelis–Menten, hyperbolic fit (*P* < 0.001). Additionally, exemplary sigmoidal/Hill and Michaelis–Menten fits for the smaller substrate increments are presented in the insets for Figures 1C and 2A. These insets represent the difference between both models, demonstrating the sigmoidal/Hill model is a better fit. A Ha/sHc glucose transport system was demonstrated, which saturated around 30–40 mmol/L glucose and resulted in a similarly low *K*_m_ and a significantly higher *V*_max_ compared to the glucose transport system in the jejunum (*K*_m_: *P* = 0.1, *V*_max_: *P* < 0.001; Table 2 and Fig. 1C). Specifically, ileal glucose transport kinetics had 9 times higher capacity than jejunal glucose transport kinetics (Table 2; compare ileum: 100.7 ± 9.3 *μ*A/cm^2^ vs. jejunum: 11.1 ± 2.3 *μ*A/cm^2^, *P* < 0.001).

The galactose‐induced gradient in the ileum resulted in a La/Hc transport kinetic system, becoming saturated around 40–50 mmol/L, with a significantly higher *K*_m_ and a lower *V*_max_ compared to glucose transport (Tables 2 and 3, and Fig. 2A, *V*_max_: *P* = 0.02 for both substrates, *K*_m_: *P* < 0.001 for both substrates). Ileal glucose transport had a significantly higher *V*_max_ than ileal galactose transport, resulting in sHc and Hc kinetics, respectively (Tables 2 and 3). Sodium dependency of glucose transport in the ileum was demonstrated by replacement of sodium with choline creating a sodium‐free Krebs buffer, which eliminated the glucose stimulated current (Fig. 1C and D).

#### Distal colon – lack of electrogenic monosaccharide transport

The addition of glucose and galactose in the colon did not generate any observable sodium‐dependent saturation kinetics, preventing the calculation of *V*_max_ or *K*_m_ values (Tables 2 and 3, Figs. 1E and 2A).

### Inhibition of glucose and galactose transport systems

#### Jejunum – inhibition of Ha/sLc glucose and La/Lc galactose systems

**Figure 3:**
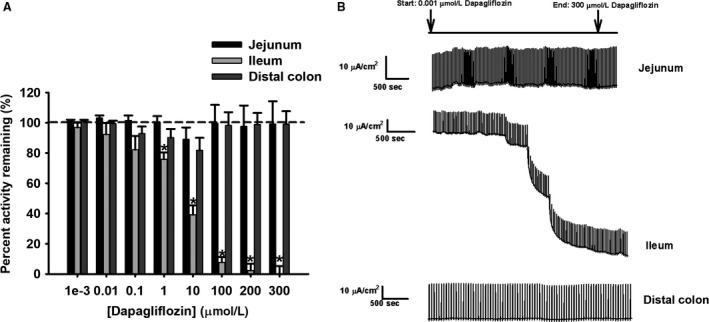
Percent activity remaining of short‐circuit current (Isc) in response to d‐glucose transport by (A) dapagliflozin (0.001, 0.01, 0.1, 10, 100, 200, and 300 *μ*mol/L) in pig intestinal tissues (jejunum: *N* = 12, ileum: *N* = 12, distal colon: *N* = 11). (B) Representative traces for all intestinal tissues are presented for illustration purposes. Data are represented as means ± SEM. Asterisks represent significance from 100% transporter activity before inhibition for the ileum, which is presented as the dotted line (one‐way ANOVA,* P* < 0.05).

**Figure 4:**
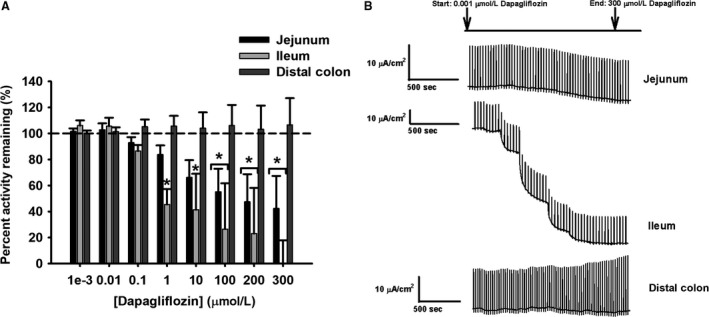
Percent activity remaining of short‐circuit current (Isc) in response to d‐galactose transport by (A) dapagliflozin (0.001, 0.01, 0.1, 10, 100, 200, and 300 *μ*mol/L) in pig intestinal tissues (all tissues: *N* = 6). (B) Representative traces for all intestinal tissues are presented for illustration purposes. Data are represented as means ± SEM. Asterisks represent significance from 100% transporter activity before inhibition for the jejunum and ileum, which is presented as the dotted line (one‐way ANOVA,* P* < 0.05).

Dapagliflozin, a selective inhibitor for SGLT2 (sodium/glucose cotransporter 2) was used, and did not inhibit glucose‐induced electrogenic current in the porcine jejunum (Fig. 3A and B) (Ferraris and Diamond 1986; Komoroski et al. 2009). Interestingly, dapagliflozin did cause significant inhibition (about 20–25%) of the galactose‐induced electrogenic current at the higher concentrations (100–300 *μ*mol/L) (Fig. 4A and B, *P* < 0.001). The *K*_*i*_ value for inhibition of jejunal galactose transport by dapagliflozin is presented in Table 4.

**Table 4:** *K*_*i*_ values for d‐glucose and d‐galactose electrogenic absorption

d‐glucose		d‐galactose	
Tissue	*K*_*i*_ ± SEM (*μ*mol/L)	Tissue	*K*_*i*_ ± SEM (*μ*mol/L)
Jejunum	nd	Jejunum	4.6 ± 0.9^**A**^
Ileum	6.0 ± 1.0	Ileum	1.8 ± 0.6^**B**^
Distal colon	nd	Distal colon	nd

*K*_*i*_ (*μ*mol/L) values represent dapagliflozin inhibition of sodium‐dependent electrogenic d‐glucose (jejunum: *N* = 12, Ileum: *N* = 12, distal colon: *N* = 11) and d‐galactose (all tissues: *N* = 6) absorption for each tissue type in pig. ND represents nondetectable inhibition. Data are presented as means ± SEM. Significant differences represented by different letter superscripts between *K*_*i*_ values for jejunum and ileum inhibition of d‐galactose transport (Student's *t*‐test, *P* < 0.05).

#### Ileum – inhibition of Ha/sHc glucose and La/Hc galactose transport systems

In the ileum, dapagliflozin significantly inhibited glucose sodium‐dependent electrogenic current from 1 to 300 *μ*mol/L dosages, resulting in 20–90% inhibition (Fig. 3A and B, *P* < 0.05). Similarly, in the galactose‐induced electrogenic current, dapagliflozin significantly inhibited from 1 to 300 *μ*mol/L dosages, resulting in approximately 40–80% inhibition (Fig. 4A and B, *P* < 0.001). The *K*_*i*_ values for inhibition of ileal glucose and galactose transport are presented in Table 4. Finally, the significant difference in *K*_*i*_ values observed between jejunal and ileal galactose inhibition, as well as the absence of inhibition in jejunal glucose transport, suggest different multiple transporter populations between segments (Table 4).

#### Distal colon – lack of inhibition of monosaccharide transport

A lack of inhibition with dapagliflozin for glucose‐ and galactose‐induced electrogenic currents confirmed the nondetectable kinetic transport system (Figs. 3A and 4A).

### Genomic and gene expression analysis

#### SLC5A gene identification affirms kinetics in the jejunum

To identify gene product candidates that could contribute to the observed kinetics in the porcine jejunum, BLAST+ was utilized to probe the known SLC5A gene family. Our findings revealed 12 porcine SLC5A members in the genome (Table 1). However, quantitative real‐time PCR only identified 10 porcine SLC5A members in the intestine. The SLC5A transporters identified with qPCR were SGLT1 (SLC5A1, sodium/glucose cotransporter 1), SGLT2 (SLC5A2, sodium/glucose cotransporter 2), SMIT1 (SLC5A3, sodium/myoinositol cotransporter 1), SGLT3 (SLC5A4, sodium/glucose cotransporter 3), SMVT (SLC5A6, sodium/multivitamin cotransporter), CHT (SLC5A7, sodium/choline cotransporter), SMCT1 (SLC5A8, sodium/monocarboxylate cotransporter 1), SGLT5 (SLC5A10, sodium/glucose cotransporter 5), SMIT2 (SLC5A11, sodium/myoinositol cotransporter 2), and SMCT2 (SLC5A12, sodium/monocarboxylate cotransporter 2). The genes NIS (SLC5A5, sodium/iodide cotransporter) and SGLT4 (SLC5A9, sodium/mannose cotransporter) were not detected by qPCR in the pig jejunum.

**Figure 5:**
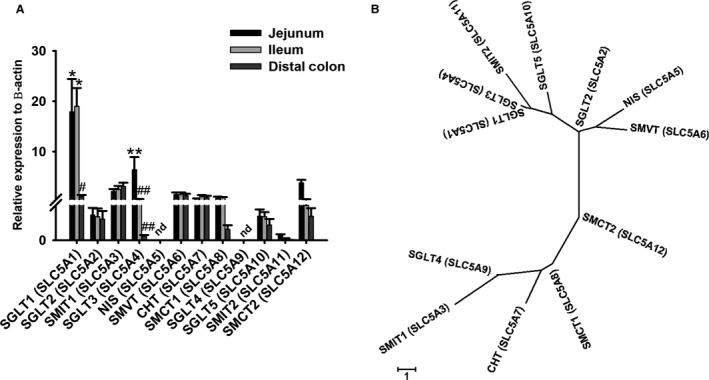
(A) Relative expression levels of SLC5A transporters to *β*‐actin (housekeeping gene) (jejunum: *N* = 8–10, ileum: *N* = 7–10, distal colon: *N* = 7–10) using quantitative PCR. The expression levels of each gene were determined from the difference with the *β*‐actin housekeeping gene. Significant differences were found between genes SGLT1 (SLC5A1) and the rest of the members in the jejunum and ileum, represented by single asterisks (two‐way ANOVA,* P* < 0.05). Significant differences were found between SGLT1 (SLC5A1) in the jejunum and ileum versus distal colon, represented by single asterisks and single number sign (two‐way ANOVA,* P* < 0.05). Significant differences found with SGLT3 (SLC5A4) between the jejunum versus ileum and distal colon, represented by double asterisks and double number signs (two‐way ANOVA,* P* < 0.05). ND represents nondetectable expression of those genes. (B) Phylogenic tree of the 12 members of the SLC5A family of cotransporters in pig. The alignment program CLUSTAL W and the phylogenic display program MEGA7 were used to generate the tree.

More specifically, porcine SGLT1 (SLC5A1) expression was significantly greater than the other nine identified SLC5A isoforms in the jejunum, supporting the Ha/sLc glucose and La/Lc galactose transport systems (Fig. 5A, *P* < 0.001 for transporter type). In addition, SGLT3 (SLC5A4) expression in the jejunum was significantly greater than the expression in the ileum and distal colon (Fig. 5A, *P* < 0.05 for transporter type). Both gene candidates support the observed kinetics and inhibitor data found in the porcine jejunum. Sequence similarities between pig SLC5A transporters were compared in a phylogenic tree (Fig. 5B). In a CLUSTAL W analysis, the nucleotide identities relative to pig SGLT1 (SLC5A1) were 57.5% SGLT2 (SLC5A2), 48.1% SMIT1 (SLC5A3), 72.8% SGLT3 (SLC5A4), 40.3% NIS (SLC5A5), 39.8% SMVT (SLC5A6), no sequence similarity with CHT (SLC5A7), 37.3% SMCT1 (SLC5A8), 51.7% SGLT4 (SLC5A9), 56.6% SGLT5 (SLC5A10), 56.1% SMIT2 (SLC5A11), and 35.3% SMCT2 (SLC5A12), revealing highest nucleotide sequence similarity between SGLT1 (SLC5A1) and SGLT3 (SLC5A4).

#### SLC5A gene profiling in the ileum

To identify gene product candidates that could contribute to the observed kinetics in the porcine ileum, BLAST+ was utilized to probe the known SLC5A gene family. Similar to the findings in the jejunum, only 10 SLC5A members were identified by qPCR in the ileum, with no detection of NIS (SLC5A5) and SGLT4 (SLC5A9).

Gene expression in the ileum revealed significantly greater expression of SGLT1 (SLC5A1) when compared to the other nine SLC5A isoforms (Fig. 5A, *P* < 0.001). In contrast to the jejunum, SGLT3 (SLC5A4) expression was not significantly different between the ileum and distal colon (Fig. 5A). Sequence similarities between pig SLC5A transporters were compared in a phylogenic tree (Fig. 5B). Overall, sigmoidal kinetics of monosaccharide transport demonstrated in the ileum raises the possibility of another transporter in addition to SGLT1 (SLC5A1), which is either a modified SGLT1 (SLC5A1) different than the jejunum, or another transporter.

#### SLC5A gene profiling in the distal colon

Identification of 10 SLC5A transporters in the distal colon by qPCR resulted in low expression of all genes in comparison to the jejunum and ileum, supporting the absence of electrogenic monosaccharide transport in this segment (Fig. 5A).

## Discussion

In terms of porcine sodium‐dependent glucose transport, our results mirror studies presented previously that represent an overall low‐capacity of transport in the jejunum and an overall high‐capacity of transport in the ileum (Halaihel et al. 1999; Herrmann et al. 2012). In addition to these studies, we have presented sodium‐dependent galactose transport following a similar pattern of low‐capacity jejunal transport and high‐capacity ileal transport, which has not been characterized before. However, in contrast to previous studies, this study presents both glucose and galactose sodium‐dependent transport following sigmoidal/Hill kinetic systems, instead of previously characterized Michaelis–Menten systems (Halaihel et al. 1999; Herrmann et al. 2012). This kinetic characterization of glucose and galactose transport was demonstrated by both the jejunum and ileum. Therefore, this strongly suggests the involvement of multiple transporter populations contributing to the kinetic systems observed in each segment, which is contrary to previous studies (Halaihel et al. 1999; Herrmann et al. 2012). Finally, pharmacological inhibition and gene expression analysis support the presence of different multiple transporter populations in each segment.

### Jejunum

#### Sigmoidal kinetics support multiple transporter involvement in the jejunum

In this study, the porcine jejunum electrogenic glucose absorption is defined as a Ha/sLc system following sigmoidal/Hill kinetics, which becomes saturated at 14–15 mmol/L glucose (Fig. 1A, and Tables 2 and 3). Comparatively, jejunal galactose transport revealed a La/Lc system, with 4 times lower affinity than glucose transport (Tables 2 and 3). Previous studies that have characterized glucose transport systems revealed a Ha/Lc transport system following a Michaelis–Menten model (Brot‐Laroche et al. 1988; Halaihel et al. 1999; Kellett and Helliwell 2000; Wood and Trayhurn 2003; Wright et al. 2004, 2011; Kanwal et al. 2012). The use of Michaelis–Menten fits in these studies may be due to a smaller number of glucose increments used (Brot‐Laroche et al. 1988; Halaihel et al. 1999; Kellett and Helliwell 2000; Wood and Trayhurn 2003; Wright et al. 2004, 2011; Kanwal et al. 2012). However, the results in this study reveal both glucose and galactose transport following a sigmoidal/Hill fit, which is represented by an “S”‐shaped curve, rather than a hyperbolic curve (Figs. 1A and 2A). Additionally, we provided exemplary fits between the sigmoidal/Hill and Michaelis–Menten models for jejunal glucose and galactose transport, which further emphasized that the sigmoidal/Hill model is a better fit (Figure insets 1A and 2A). In comparison to previously described Michaelis–Menten kinetics, the sigmoidal/Hill fits demonstrated here may be due to the techniques used, the increased number of glucose increments, as well as a larger glucose/galactose range (Figs. 1 and 2). Nonetheless, with both jejunal glucose and galactose transport following sigmoidal/Hill fits, it strongly suggests the possibility of multiple transporter populations contributing to these kinetic transport systems.

Pharmacological inhibition with dapagliflozin lacked effect on jejunal sodium‐dependent glucose transport, but inhibited jejunal galactose transport at concentrations greater than 100 *μ*mol/L (Figs. 3A and 4A, respectively). The lack of inhibition on glucose transport is not surprising given the low expression of SGLT2 (SLC5A2) in the jejunum, since dapagliflozin is a specific, competitive inhibitor for SGLT2 (SLC5A2) (Fig. 5A) (Komoroski et al. 2009; Obermeier et al. 2010; Isaji 2011; Hummel et al. 2012). In contrast, the inhibition of galactose‐induced transport by dapagliflozin may be explained by the lower affinity for galactose by a transporter such as SGLT1 (SLC5A1), thus allowing for competitive inhibition by dapagliflozin. Additionally, the higher increment concentrations of dapagliflozin used in this study may have contributed to an inhibitory effect on SGLT1 (SLC5A1) for jejunal galactose transport, where previously it has been shown dapagliflozin can inhibit SGLT1 (SLC5A1) starting at 1 *μ*mol/L (Hummel et al. 2012). Alternatively, it may also suggest the presence of another transporter such as SGLT3 (SLC5A4) that may be sensitive to dapagliflozin inhibition with galactose transport.

Following inhibitor characterization, a genomic and gene expression analysis of all porcine SLC5A genes revealed that SGLT1 (SLC5A1) and SGLT3 (SLC5A4) had the greatest expression in the porcine jejunum (Fig. 5A). The high SGLT1 (SLC5A1) expression mirror those from previous studies that found SGLT1 (SLC5A1) as the dominant ortholog in guinea pig and porcine jejunal BBMVs, supporting a Ha/Lc system in this segment (Brot‐Laroche et al. 1988; Halaihel et al. 1999; Kellett and Helliwell 2000; Wood and Trayhurn 2003; Wright et al. 2004, 2011; Kanwal et al. 2012; Klinger et al. 2018). Additionally, studies in rats, mice, horses, rabbits, and beef steers found that SGLT1 (SLC5A1) expression was greater in the jejunum than ileum, which affirmed SGLT1 (SLC5A1) as a contributor to the Ha/Lc system (Brot‐Laroche et al. 1988; Halaihel et al. 1999; Kellett and Helliwell 2000; Wood and Trayhurn 2003; Wright et al. 2004, 2011; Herrmann et al. 2012; Kanwal et al. 2012). SGLT1 (SLC5A1) is known to absorb both glucose and galactose, but with a lower affinity for galactose (Wright 2013).

However, the sigmoidal kinetic systems for jejunal glucose and galactose transport, strongly suggest the presence of multiple transporter populations in this segment. Therefore, the high expression of SGLT3 (SLC5A4) may be the other transporter involved in the jejunum (Fig. 5A). In pigs, SGLT3 (SLC5A4) was first identified as SAAT1 (low‐affinity sodium‐dependent amino acid transporter) in the porcine kidney, which was mistakenly identified as SGLT2 in the porcine intestine, and finally confirmed as SGLT3 (SLC5A4) as a low‐affinity glucose transporter (Diez‐Sampedro et al. 2001; Wood and Trayhurn 2003; Wright 2013). It has a lower affinity for glucose and galactose compared to SGLT1 (SLC5A1) (Diez‐Sampedro et al. 2001). Overall, SGLT1 (SLC5A1) and SGLT3 (SLC5A4), or secondary modifications of these transporters, may contribute to the sigmoidal Ha/sLc and La/Lc glucose and galactose transport systems, respectively, further supporting the notion of multiple transporter populations in the porcine jejunum.

### Ileum

#### Multiple transporter populations involved in ileal monosaccharide transport which differ from the jejunum

A Ha/sHc glucose transport system following sigmoidal/Hill kinetics was characterized in the porcine ileum with significant differences in kinetics and pharmacological inhibition compared to the jejunum. The kinetics revealed a similar affinity, but a nine times higher capacity in the ileum compared to jejunal glucose transport (Tables 2 and 3). Previous studies comparing glucose transport between porcine jejunal and ileal BBMVs found that the ileum had a higher *V*_max_ than the jejunum, but similar *K*_m_ values, supporting findings in the current study (Halaihel et al. 1999; Herrmann et al. 2012). For the first time, our study also revealed sodium‐dependent galactose transport had a La/Hc kinetic system following sigmoidal/Hill kinetics in the ileum, but at a higher capacity than the jejunum (Tables 2 and 3). Similar to the jejunum, the sigmoidal/Hill kinetics for glucose and galactose transport in the ileum strongly suggest the presence of multiple transporter populations contributing to these kinetic systems. The sigmoidal/Hill kinetics in the ileum also follows an “S”‐shaped curve rather than a hyperbolic curve, similar to the jejunum. Additionally, we provided exemplary fits between the sigmoidal/Hill and Michaelis–Menten models for ileal glucose and galactose transport, which demonstrated that the sigmoidal/Hill model is a better fit (Figure insets 1C and 2A). Finally, pharmacological inhibition and gene expression analysis further support these results.

Dapagliflozin inhibited both glucose and galactose transport at concentrations 1–300 *μ*mol/L in the ileum (Figs. 3A and 4A). These results are different from the jejunum, where dapagliflozin inhibition was only observed with jejunal galactose transport. Here, inhibition by dapagliflozin for ileal glucose and galactose transport decreased transporter activity close to basal levels. This effect has been previously described with phloridzin dihydrate (Herrmann et al. 2012). Phloridzin was shown to have a greater inhibitory effect in the ileum than the jejunum with glucose transport, decreasing Isc to almost basal levels in the ileum (Herrmann et al. 2012). Therefore, the difference in dapagliflozin inhibition between the jejunum and ileum mirrors the results seen with inhibition by phloridzin, further emphasizing the difference in transporters and transport systems between these intestinal segments. Additionally, dapagliflozin is a selective inhibitor for SGLT2, which is specifically a La/Hc transporter in the kidney (Komoroski et al. 2009; Chao and Henry 2010; Isaji 2011; Hummel et al. 2012). The greater inhibitory effect of dapagliflozin on ileal glucose and galactose transport compared to the jejunum is reasonable, since the ileum represents an overall Hc system for both monosaccharides, even if it is not contributed by SGLT2 (SLC5A2). Additionally, compared to jejunal galactose inhibition, the *K*_*i*_ value for ileal galactose inhibition is significantly smaller (Table 4). The higher sensitivity to dapagliflozin inhibition with galactose transport in the ileum compared to the jejunum further supports the kinetic transport differences between the jejunum and ileum. Finally, the higher concentration increments of dapagliflozin may have contributed to a strong inhibitory effect with glucose and galactose transport in the ileum (Hummel et al. 2012). Therefore, this strongly suggests the presence of another transporter in the ileum that is not similar to either SGLT1 (SLC5A1) or SGLT3 (SLC5A4) identified in the jejunum due to different patterns of inhibition.

Gene expression analysis determined that SGLT1 (SLC5A1) was the dominant SLC5A ortholog expressed in the porcine ileum. This is similarly described in other mammalian species such as rabbits, mice, and rats, where the high expression of SGLT1 (SLC5A1) was found in the ileum (Koepsell and Spangenberg 1994; Halaihel et al. 1999; Herrmann et al. 2012, 2016; Vrhovac et al. 2015). These findings suggest that SGLT1 (SLC5A1) may contribute to the Ha glucose transport system and the La galactose transport system in the porcine ileum. This is supported by previous studies indicating SGLT1 (SLC5A1) has a higher affinity for glucose than galactose (Wright 2013). Additionally, the dapagliflozin inhibitor may also inhibit the SGLT1 (SLC5A1) transporter present in this segment, where dapagliflozin has been previously reported to inhibit SGLT1 (SLC5A1) starting at 1 *μ*mol/L concentration (Hummel et al. 2012). With the higher increment concentrations of dapagliflozin used in this study, this is a possibility. However, SGLT1 (SLC5A1) alone does not explain the sHc and Hc kinetics observed with glucose and galactose transport, as well as the differences in dapagliflozin inhibition to the glucose‐ and galactose‐induced currents between the jejunum and ileum. Additionally, it has been concluded previously that SGLT1 (SLC5A1), although having dominant expression in the ileum, was not wholly responsible for the overall Hc glucose transport in the ileum (Herrmann et al. 2012). Thus, suggesting more than one transporter involved in ileal glucose transport, although only Michaelis–Menten kinetics were found (Herrmann et al. 2012). Here, more importantly, the sigmoidal/Hill fits observed with ileal monosaccharide transport strongly suggest the involvement of another transporter. Finally, the contribution of the other SLC5A isoforms to the transport kinetics observed in the ileum are insignificant when compared with their similarly low expression in the distal colon, which lacks electrogenic monosaccharide transport (Fig. 5A).

### Distal colon

#### Lack of electrogenic monosaccharide transport

In this study, the distal colon represents a negative control, used to demonstrate a lack of electrogenic glucose and galactose transport in comparison to the jejunum and ileum. Our findings are supported by previous studies that have suggested that the colon lacks glucose transport (Pácha 2000; Wood and Trayhurn 2003). During neonatal development in pigs, mice, and rats, the colon possess villus‐like structures that assist in carbohydrate absorption, but this property disappears soon after birth (Pácha 2000). The pigs used in the current study were weaned and 7–9 weeks of age, thus having a mature colon lacking monosaccharide absorptive properties, producing the observed results (Figs. 1E and 2A). Additionally, the lack of dapagliflozin inhibition and the poor expression of all SLC5A genes further support the absence of sodium‐dependent glucose and galactose transport in the colon.

### Limitations

There are a few limitations to address in this study. The direct correlation between mRNA and protein expression does not always hold. Therefore, the identification of the porcine SLC5A members through RT‐qPCR does not confirm whether the protein expression is also present. However, to perform western blot analysis to confirm protein expression is not possible with no porcine antibodies available for all of the identified SLC5A isoforms. Even if antibodies were available for protein detection, the transporters identified would only represent an association with the kinetics observed. Another limitation addresses whether SGLT3 (SLC5A4) possibly plays a role in the sigmoidal kinetics observed in the jejunum. Performing experiments to further verify whether SGLT3 is responsible for the kinetics in the jejunum (porcine SGLT3 knockout) would be beyond the scope of this study. Additionally, experiments involving the cloning and expression of porcine SGLT3 would still not confirm whether SGLT3 is the other transporter responsible for the sigmoidal kinetics. Finally, different transporters expressed in the jejunum and possibly the ileum may not necessarily mean different transporters are responsible for the kinetic systems observed. Another possibility may be secondary modifications to the other SLC5A transporters identified by the gene expression in certain situations.

## Conclusion

Electrogenic sodium‐dependent glucose and galactose absorption in the jejunum and ileum followed sigmoidal/Hill kinetics, suggesting the presence of multiple transporter populations in each segment. In support of this, the jejunum had dominant expression of SGLT1 (SLC5A1) and SGLT3 (SLC5A4), possibly contributing to the Ha/sLc glucose and the La/Lc galactose transport systems. In contrast, the ileum presents itself with Ha/sHc glucose and La/Hc galactose transport systems, with dominant expression of SGLT1 (SLC5A1). However, the sigmoidal kinetics in the ileum likely suggests the involvement of another transporter along with SGLT1 (SLC5A1). Additionally, pharmacological inhibition demonstrates differences in inhibition patterns between the jejunum and ileum supporting different multiple transporter populations in each segment. Finally, this detailed kinetic analysis provides a substantial burden of proof for not one but multiple transporter populations that differ between the jejunum and ileum in the electrogenic absorption of glucose and galactose not previously reported.

## Conflict of Interest

No conflicts of interest, financial or otherwise, are declared by the authors.
